# Domain Organization of Long Signal Peptides of Single-Pass Integral Membrane Proteins Reveals Multiple Functional Capacity

**DOI:** 10.1371/journal.pone.0002767

**Published:** 2008-07-23

**Authors:** Jan A. Hiss, Eduard Resch, Alexander Schreiner, Michael Meissner, Anna Starzinski-Powitz, Gisbert Schneider

**Affiliations:** Centre for Membrane Proteomics, Institute of Cell Biology and Neuroscience, Goethe-University, Frankfurt am Main, Germany; Max Planck Institute for Evolutionary Anthropology, Germany

## Abstract

Targeting signals direct proteins to their extra - or intracellular destination such as the plasma membrane or cellular organelles. Here we investigated the structure and function of exceptionally long signal peptides encompassing at least 40 amino acid residues. We discovered a two-domain organization (“NtraC model”) in many long signals from vertebrate precursor proteins. Accordingly, long signal peptides may contain an N-terminal domain (N-domain) and a C-terminal domain (C-domain) with different signal or targeting capabilities, separable by a presumably turn-rich transition area (tra). Individual domain functions were probed by cellular targeting experiments with fusion proteins containing parts of the long signal peptide of human membrane protein shrew-1 and secreted alkaline phosphatase as a reporter protein. As predicted, the N-domain of the fusion protein alone was shown to act as a mitochondrial targeting signal, whereas the C-domain alone functions as an export signal. Selective disruption of the transition area in the signal peptide impairs the export efficiency of the reporter protein. Altogether, the results of cellular targeting studies provide a proof-of-principle for our NtraC model and highlight the particular functional importance of the predicted transition area, which critically affects the rate of protein export. In conclusion, the NtraC approach enables the systematic detection and prediction of cryptic targeting signals present in one coherent sequence, and provides a structurally motivated basis for decoding the functional complexity of long protein targeting signals.

## Introduction

Targeting signals are contiguous stretches of amino acids that direct proteins to their sub-cellular destinations or the extracellular space [1]. With few exceptions, the vast majority of extracellular proteins are exported from mammalian cells *via* the endoplasmic reticulum (ER) secretory pathway [2]. While most signal sequences are N-terminally located, deviant examples have been reported with internal signals like in human UDP-glucuronosyltransferase [3], or bacterial C-terminal secretion signals like in virulence factor from *Mycobacterium tuberculosis* [4], and *Escherichia coli* (*E. coli*) haemolysin [5].

Canonical N-terminal signals are processed by signal peptidases [6]. The sequence similarity among these cleavable “signal peptides” coding for the ER and subsequent protein export is low as they do not share common residue motifs but rather possess common physicochemical features coding for the appropriate cellular compartment [7], [8]. Signal recognition by the cellular decoding machinery may include multiple recognition events [9], [10]. This renders perfect *in silico* prediction of subcellular locations and the detection of targeting signals still impossible although many encouraging attempts have been made [11]–[16]. For example, to counter the dissimilarity in signal peptides for prediction processes, the amino acid composition has been taken into account resulting in improved accuracy [8], [17], [18]. Despite their dissimilarity, N-terminally located targeting sequences are sometimes interchangeable between proteins in eukaryotes and even between different kingdoms. One such example is *Escherichia coli* (*E. coli*) beta-lactamase, which can be exported by *Xenopus* oocytes [19]. Still, general signal interchangeability cannot be postulated [20], [21]. Public web servers are available for predicting the subcellular localization of proteins in various organisms, for example *Cell-PLoc* (http://chou.med.harvard.edu/bioinf/Cell-PLoc/) [22] or the *SignalP* suite (http://www.cbs.dtu.dk/services/SignalP/) [14].

In eukaryotes, a canonical N-terminally located protein export signal typically contains three distinguishable parts: a positively charged N-terminal section (*n*-region), a hydrophobic core (*h*-region), and a signal peptidase recognition site (*c*-region) [8], [11]. The approximate average length of such signal peptides is 22 amino acid residues [23]. While the *c*-region typically consists of five residues, both the *h*- and the *n*-region show more variability in length. This variability has been suggested to enable alternative functions [10], [24]. In fact, much longer examples of signal peptides are known to exhibit additional functions besides precursor targeting [10], [25], [26], for example regulation of the protein export rate as described for interleukin-15 [27], or signal peptide accumulation in the nucleoli in the case of mouse mammary tumor virus Rem protein after release from the endoplasmic reticulum [28].

In the present study, we introduce a structurally motivated modularization of long signal peptides into separate functional modules, and demonstrate the actual functional relevance of this concept for the long signal peptide of the integral membrane protein shrew-1 (SH) as an example. Shrew-1 was originally isolated from an epithelial-like cell line obtained from an endometriosis biopsy [29]. It contains a cleavable N-terminal signal peptide of 43 residues [30], an extracellular domain (residues 44–282), a transmembrane segment (residues 283–303) and a cytoplasmic domain (residues 304–411). Shrew-1 is transported to the basolateral part of the plasma membrane in polarized epithelial cells and interacts with the E-cadherin mediated adherens junction complex [29], [31]. In nonpolarised cells, like transformed epithelial cells, shrew-1 also displays plasma membrane localization, though apparently less polarized. Shrew-1 appears to be involved in the regulation of cell invasion and motility and, in line with this, interacts with protein CD147, a known promoter of invasiveness [32].

Based on proteome analysis by machine-learning systems, we propose a bipartite domain model (“NtraC” model) of long signal peptides from single-pass integral membrane proteins. According to this model, such long signal peptides may contain two separate functional domains: an N-terminal domain (“N-domain”) and a C-terminal domain (“C-domain”) traceable by a turn-rich linker area connecting both. We denote this linker element “transition area” (tra). Proof-of-principle for the validity of the NtraC domain model is provided by *in vitro* targeting experiments with shrew-1.

## Results

### Many single-spanning integral membrane proteins possess long signal peptides with a bipartite domain organization

Analysis of long signal peptides was performed in two steps: First, potential domains were predicted using a novel machine-learning technique for turn prediction [33]. Potential turn-containing regions were found to be predominantly located in the central portion of these long signals. Based on the location of this “transition area”, long signal peptides were dissected into two parts, an N-terminal (‘N’) and a C-terminal (‘C’) fragment. Then, the resulting sequence fragments were scrutinized for potential targeting functions. The concept of this NtraC model of signal peptide organization is based on the hypothesis that the two functional modules in a long signal peptide may exhibit individually distinct tasks in the context of protein targeting. This requires a minimal peptide length, and for the present study we decided to focus only on signal peptide domains containing conventional signals with an expected average length of approximately 20 residues each. This choice is motivated by the observed average length of targeting signals coding for a single compartment [23]. Certainly, we cannot exclude the existence of other targeting signals of hitherto unknown structure (*e.g.*, unusually short signals) within long signal peptides.

Searching for long signal peptides (≥40 residues) in the UniProtKB database (release 53.2) [34] yielded 296 vertebrate proteins, including homologues. All sequences were analyzed with regard to their potential NtraC organization. Within our NtraC analysis software, predictions for potential targeting signals were done using the software *SignalP 3.0* [23] (signals coding for protein transport into the ER, signal peptide and signal anchor prediction) and *TargetP* [35] (signals coding for mitochondrial import). Potential turn-forming elements were detected using our software tool SVMTurn (www.modlab.deSoftwareSVMTurn) [33]. SVMTurn uses Support Vector Machine classifiers for recognition of various turn types in amino acid sequences. Turns with intramolecular hydrogen bonds encompassing four, five, and six residues are predicted with approximately 80% accuracy.

**Figure 1:**
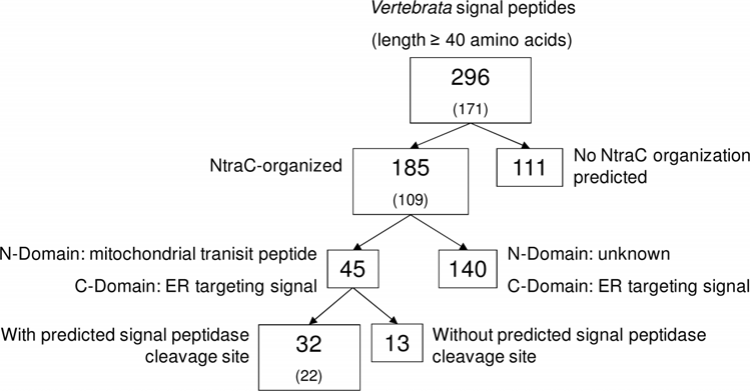
Overview of NtraC-organized sequences among long signal sequences found in vertebrate proteins. Set sizes without orthologues are given in brackets. The numbers represent conservative estimates based on validated prediction tools for targeting signal recognition and turn structure prediction.

According to NtraC (www.modlab.deSoftwareNtraC) analysis, 185 of 296 (62%) long signal peptides obey the NtraC domain organization with a C-domain coding for an ER targeting signal (Suppl. Table S1). We found no strict conservation of turn residues in all 185 sequences. As expected for beta-turns, Gly is overrepresented at residue position 3 of a regular beta turn [36]. 45 of thee 185 candidate proteins possess both an N-domain coding for a putative mitochondrial transit peptide and a C-domain coding for an endoplasmic reticulum (ER) targeting signal (Figure 1). For 13 of these sequences, signal peptidase cleavage sites were not predicted. Thus, they might act as signal anchors. All 32 remaining candidates, which show a predicted domain combination analogous to shrew-1 (N-Domain: mTP, C-domain: SP) and posses a predicted signal peptidase cleavage site, are listed in Table 1. The C-domains of the remaining 140 NtraC-organized sequences code for ER targeting. In contrast to shrew-1, however, their N-domains may contain an additional feature or targeting function that is different from conventional mitochondrial targeting signals.

**Table 1:** 32 Vertebrate signal peptides >40 amino acids, which are predicted to be NtraC organized and are similar in their domain capacity to shrew-1.

ID	NCBI Accession Number	Signal peptide sequence
1	P70505	MSVAASASRSASTLCSPQIQQGALKEAKVPPHIWAARHWNLGLRLVPGHASVRAGILVLLIFLPSTLC
2	P17405	MPRYGASLRQSCPRSGREQGQDGTAGAPGLLWMGLVLALALALALA
3	Q96PD2	MASRAVVRARRCPQCPQVRAAAAAPAWAALPLSRSLPPCSNSSSFSMPLFLLLLLVLLLLLEDAGA
4	Q91ZV3	MASRAPLRAARSPQGPGGPAAPAATGRAALPSAGCCPLPPGRNSSSRPRLLLLLLLLLQDAGG
5	Q91ZV2	MASRAPLRAARSPQDPGGRAAPAATGRAPLPSAGWCPLPPGRNSSSRPRLLLLLLLLLPDAGA
6	Q28110	MGIPSFLAFPAARRNRAHCTPWHPWGHMLLWTALLFLAPVSG
7	Q1LZH9	MRLLSLAPDRPRRGGPRHLTSGSPALPPPPPLLLLLLLLGGCLGVSGA
8	P50426	MRFLSLAPDRPRRGGPRHLPSGSPAPPPPPPLLLLLLLGGCLGVSGA
9	P52785	MSAWLLPAGGLPGARFCVPARQSPSSFSRVLRWPRPGLPGLLLLLLLPSPSALS
10	P51840	MSAWLLPAGGFPGAGFCIPAWQSRSSLSRVLRWPGPGLPGLLLLLLLPSPSAFS
11	P51841	MFLGLGRFSRLVLWFAAFRKLLGHHGLASAKFLWCLCLLSVMSLPQQVWT
12	Q8K201	MAASALGRMCGAAREKLSPGPGARGLGALARSLVLALLLVPVLC
13	Q5R5B8	MAAAALKRMRGPAQAKLLPGSAIQALVGLARPLVLALLLVSAALSSVVS
14	Q9UBX7	MQRLRWLRDWKSSGRGLTAAKEPGARSSPLQAMRILQLILLALATGLVGG
15	Q5XNR9	MMNISLRLRRPPWMVDSNGRRMTSHFQWLLLTFILLYLMNQVTS
16	Q9H0V9	MAATLGPLGSWQQWRRCLSARDGSRMLLLLLLLGSGQGPQQVGA
17	P59481	MAAASRPSWWQRWRRRAWARDGAKLLLFLLLLGSGPGPRHVRA
18	Q6VE48	MRASCTPLKAPLRRPERLASSGRFAWVLLLAPLLLLPTSSDA
19	Q8VE43	MRGAVWAARRRAGQQWPRSPGPGPGPPPPPPLLLLLLLLLGGASA
20	Q5RJL6	MRGVVWAARRRAGQQWPRSPGPGPGPPPPPPLLLLLLLLLGGASA
21	Q9R0S2	MPRSRGGRAAPGQASRWSGWRAPGRLLPLLPALCCLAAAAG
22	Q99PW6	MPRSRGGRAAPGQAARWSGWRAPGRLLPLLPALCCLAAAAG
23	P29122	MPPRAPPAPGPRPPPRAAAATDTAAGAGGAGGAGGAGGPGFRPLAPRPWRWLLLLALPAACSA
24	Q9NQS3	MARTLRPSPLCPGGGKAQLSSASLLGAGLLLQPPTPPPLLLLLFPLLLFSRLCGALA
25	Q96B86	MQPPRERLVVTGRAGWMGMGRGAGRSALGFWPTLAFLLCSFPAATSP
26	Q9N0A6	MGGPGPRRAGTSRERLVVTGRAGWMGMGRGAGRSALGFWPTLAFLLCSFPAAT
27	Q6PCX7	MQPPRERLVVTGRAGWMGMGRGAGRSALGLWPTLAFLLCSFPAAISP
28	Q9QUR8	MTPPPPGRAAPSAPRARVLSLPARFGLPLRLRLLLVFWVAAASA
29	Q9UPZ6	MGLQARRWASGSRGAAGPRRGVLQLLPLPLPLPLLLLLLLRPGAGRA
30	Q9EPU5	MGTRASSITALASCSRTAGQVGATMVAGSLLLLGFLSTITA
31	Q8IZC6	MGAGSARGARGTAAAAAARGGGFLFSWILVSFACHLASTQG
32	Q91443	MGRHSALGLSGNRQVSPCTGTRPFKVVGSRSPVQPLCILLALTVCIGTS

Underlined residues are predicted turns belonging to the transition area.

To check the influence of a potential bias in these results due to clusters of homologues in the set of 296 candidate genes, we manually eliminated all orthologues. This procedure did not affect the ratio of NtraC-organized vs. non-NtraC-organized samples (Figure 1, values in brackets). In the human genome alone, we found 105 signal peptides with ≥40 residues overall, among which 71 (68% of 105) are NtraC-organized.

We provide a public web service for NtraC analysis of amino acid sequences (www.modlab.deSoftwareNtraC) and invite the scientific community to scrutinize our NtraC domain model using this prediction server.

Proteins with NtraC-organized signal sequences apparently have common features. 19 of the 32 candidate sequences are annotated in UniProt as type-I membrane proteins containing a single potential transmembrane segment (TMS). Among these, the only experimentally validated TMS is the one of shrew-1 [29], which was a clear motivation for us to use this protein for the cellular proof-of-principle study. We then performed TMS predictions for the 13 remaining sequences using the software tools Phobius [37] and SVMtm [38], which in all cases gave rise to the same results: Two proteins yielded strong positive scores indicating the likely presence of a TMS, three received weaker scores favoring TMS presence, and eight are seemingly devoid of a TMS. These results increase the number of candidate proteins from 19 to 24 out of 32, corresponding to 75% as a conservative estimation.

Summarizing, we identified a class of long signal peptides distinguished by the NtraC domain architecture. This structural and functional organization is present in signal peptides of many single-pass membrane proteins. For further study, we selected one of these proteins, human shrew-1 as an example.

### Experimental system for assessment of prediction results: Shrew-1 signal peptide and SEAP reporter protein

Based on the theoretical analysis described in the previous paragraph, we used secreted alkaline phosphatase (SEAP) as a reporter protein in order to probe the targeting capacity of the predicted domains of shrew-1's signal peptide. The SEAP reporter system allows for the exchange of the intrinsic signal peptide by other potential signal peptide sequences, which can then be tested for biological activity [39]. SEAP is a glycoprotein which becomes N-glycosylated by oligosaccharyl transferase located in the ER [40]. Therefore, its N-glycosylation status is an indication of translocation into the ER lumen, which in turn is a prerequisite for SEAP secretion into the supernatant.

#### The C-domain acts as a secretion signal

According to the NtraC model, the shrew-1 signal peptide (residues 1–43 [30], *SignalP 3.0* probability  = 0.95) is divided into three domains: It contains an N-domain (residues 1–19) and a C-domain (residues 20–43) connected by the transition area (residues 16–24). The C-domain is predicted as a standard secretion signal containing an *n*-, *h*-, and *c*-region (*SignalP 3.0* probability  = 0.9), whereas the N-domain receives a prediction as a mitochondrial transit peptide (*TargetP* probability  = 0.3).

Within the transition area, three adjacent and partly overlapping β-turns were predicted (positions 16–24). Interestingly, no further β-turns were found in the remainder of the signal peptide. The position of the turns appears to be evolutionary conserved among different species, as shown by a multiple sequence alignment of seven vertebrate shrew-1 homologues, suggesting a fundamental functional importance of this region (Suppl. Figure S1).

**Figure 2:**
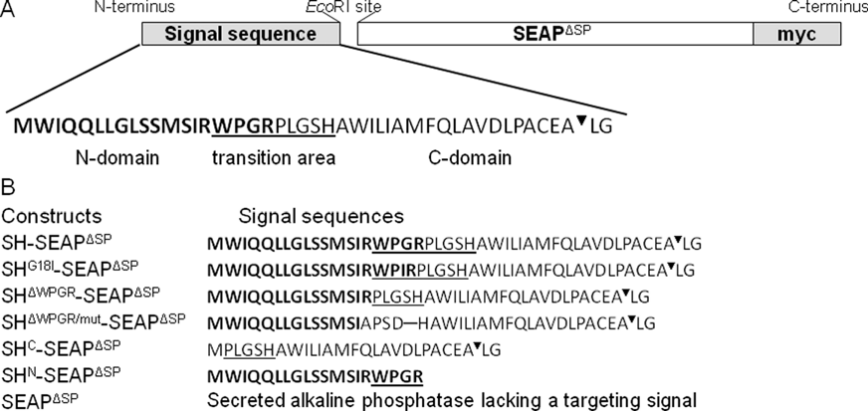
Shrew-1 (SH) signal sequence and the construction of the SEAP fusion proteins. (A) Organization of shrew-1 signal sequence. Bold: N-domain (shrew-1 residues 1–19). Standard type: C-domain (shrew-1 residues 20–43). Underlined: transition area (shrew-1 residues 16–24). ^▾^: signal sequence cleavage site. LG: shrew-1 residues 44 and 45. (B) Diagrams of SEAP constructs with assigned shrew-1 signal sequences. Signal sequences are N-terminally fused to the SEAP protein lacking the endogenous signal peptide (SEAP^ΔSP^). C-terminally, all fusion proteins are tagged with myc (EQKLISEEDL). For cleavage site recognition (PACEA^▾^LG) shrew-1 residues 44 and 45 (LG) are included in the constructs.

To functionally test the predicted signal peptide domains, six constructs coding for different SEAP fusion proteins were devised (Figure 2). They were transfected into HEK 293T cells, and SEAP activity was determined in both the supernatants and in whole cell lysates.

**Figure 3:**
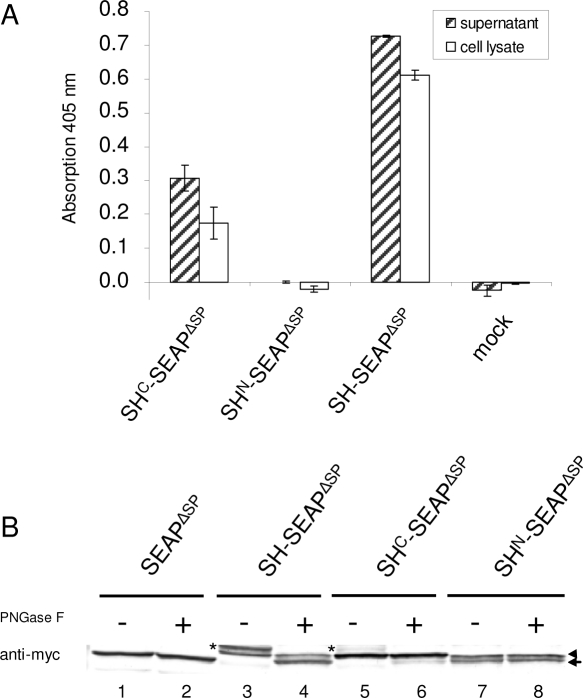
Influence of the isolated N- and C-domain on the expression, the activity and secretion of the SEAP fusion proteins. (A) SEAP activity was recorded in the supernatant (hatched bars) and whole cell lysate (white bars) of transfected HEK 293T cells after 5 minutes of substrate incubation. Cells transfected with the empty vector were used as negative control (mock). Error bars show *s.e.m.* (*N* = 4). (B) Cell lysates of HEK 293T cells expressing either SEAP^ΔSP^, SH-SEAP^ΔSP^, SH^C^-SEAP^ΔSP^ or SH^N^-SEAP^ΔSP^ were treated with PNGase F (+) or were left untreated (-) and Western blots were prepared. Fusion proteins were probed with anti-myc antibody. SH-SEAP^ΔSP^, SH^C^-SEAP^ΔSP^ or SH^N^-SEAP^ΔSP^ fusion proteins show double bands while SEAP^ΔSP^ reveals a single band which lacks N-glycosylation. SH-SEAP^ΔSP^ and SH^C^-SEAP^ΔSP^ possess an N-glycosylated protein population (*) that shifts to the position of SEAP^ΔSP^ (arrow) after PNGase F treatment. The protein population that exhibits no PNGase F sensitivity (◂) is not N-glycosylated and not N-terminally processed. SH^N^-SEAP^ΔSP^ shows no PNGase F sensitivity at all, but is also characterized by a doublet. The lower band (arrow) corresponds to the position of SEAP^ΔSP^ indicating N-terminal processing, whereas the upper band (◂) complies with the non processed protein population.

As shown in Figure 3A, the C-domain (SH^C^-SEAP^ΔSP^) alone is able to direct SEAP fusion protein to the supernatant. The N-domain (SH^N^-SEAP^ΔSP^) alone does not have this targeting capacity. The same holds for the whole cell lysates (Figure 3A, white bars).

Compared to full length shrew-1 signal peptide (SH-SEAP^ΔSP^), SEAP activity in both the supernatant and whole cell lysates of SH^C^-SEAP^ΔSP^ transfected cells was decreased to about one third. This implies that the full-length signal peptide is required for full export efficiency, but basic targeting information is encoded in the C-domain of the long signal peptide.

Notably, both fusion proteins were detectable by Western blotting (Figure 3B). This raises the question for the reason of inactivity of the N-domain containing protein. One explanation would be impaired translocation from the cytosol into the ER, which in turn should have resulted in lacking N-glycosylation of SEAP. To check this hypothesis, we subjected the lysates to PNGase F treatment, which removes N-linked glycans that are selectively found on ER-translocated active protein. Figure 3B shows that the SH^N^-SEAP^ΔSP^ protein is not N-glycosylated (lanes 7 and 8), whereas SH^C^-SEAP^ΔSP^ and SH-SESP^ΔSP^ contain an N-glycosylated SEAP population (lanes 3 and 5, band marked by an asterisk). We conclude that SH^N^-SEAP^ΔSP^ was not transported into the ER. It is noteworthy that SH^N^-SEAP^ΔSP^ was found in two non-glycosylated bands (lanes 7 and 8), indicating the existence of two populations with different molecular mass. The position of the bands is in line with the idea that the upper band contains the N-domain of the signal peptide, which might have been cleaved off in the faster migrating protein (lower band) by some non-ER protease activity.

#### The N-domain directs the reporter protein to mitochondria

**Figure 4:**
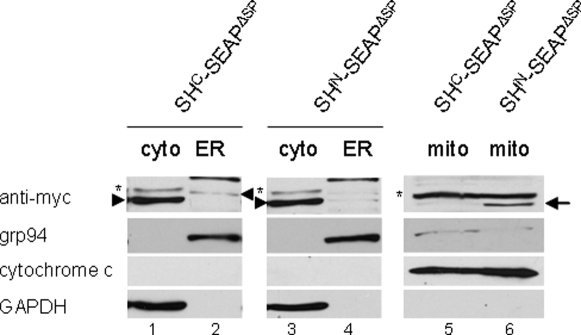
Detection of SH^N^-SEAP^ΔSP^ in mitochondria. Mitochondria were isolated from HEK 293T transfected with either SH^N^-SEAP^ΔSP^ or SH^C^-SEAP^ΔSP^, and Western blots were prepared with cytosolic (cyto), ER and mitochondrial (mito) fractions. SEAP fusion proteins were probed with antibody against the myc-tag (anti-myc). Marker proteins were grp94 for ER, cytochrome c for mitochondrial, and GAPDH for cytosolic fractions. Only SEAP fusion protein containing the N-domain of shrew-1's SP (SH^N^-SEAP^ΔSP^) was clearly detectable in the mitochondrial fraction (lane 6, arrow). Asterisks on the left indicate unspecific bands. Arrowheads mark the positions of SEAP fusion proteins in the cytosolic and ER fractions.

The observation of two non-glycosylated bands in the Western blot analysis raised the question, whether the SH^N^-SEAP^ΔSP^ fusion protein is able to target to mitochondria, as predicted by our sequence analysis (*vide supra*). Therefore, we analyzed mitochondrial localization of SH^N^-SEAP^ΔSP^. HEK 293T cells were transfected with either SH^N^-SEAP^ΔSP^ or SH^C^-SEAP^ΔSP^, and mitochondria were isolated by differential centrifugation followed by density gradient centrifugation. Cytosolic (cyto) and ER fractions obtained by differential centrifugation were positive for GAPDH as a cytosolic marker protein, or grp94 as an ER marker, and negative for cytochrome C as a mitochondrial marker (Figure 4, lanes 1–4). Mitochondria obtained by density centrifugation were completely negative for GAPDH, only a weak band corresponding to grp94 was detectable, and cytochrome C was prominently detected, indicating efficient purification of mitochondria (Figure 4, lanes 5 and 6).

SH^C^-SEAP^ΔSP^ was detectable in an unglycosylated state in the cytosolic fraction (Figure 4, lane 1) and in an N-glycosylated state in the ER fraction (Figure 4, lane 2). In contrast, it was barely detectable in the mitochondrial fraction (Figure 4, lane 5). A different distribution was found for SH^N^-SEAP^ΔSP^, which was present in the cytosolic fraction, but not in the ER fraction (Figure 4, lanes 3 and 4). This observation is in line with the absence of SEAP activity in the supernatant and whole cell lysates extracted from cells transfected with this fusion protein (Figure 3). Most importantly, SH^N^-SEAP^ΔSP^ was prominently detected in the mitochondrial fraction, which received further confirmation by immunofluorescence studies in HEK 293T cells (not shown). This experimental observation is in perfect agreement with the computational prediction.

#### Deletion of the transition area decreases secretion

The results presented so far show that the C-domain is sufficient for secretion of SEAP fusion protein, whereas the N-domain has no ER translocation capacity, but rather accommodates a mitochondrial targeting activity. However, when compared to the full length signal sequence the C-domain exhibits a decreased secretion activity. This observation gave rise to the question whether the transition area (residues 16–24) influences the efficiency of ER translocation.

To test this hypothesis, we generated constructs coding for three different SEAP fusion proteins, containing mutations and deletions in the transition area of the otherwise wild-type shrew-1 signal peptide. One contains a Gly→Ile substitution at position 18 (SH^G18I^-SEAP^ΔSP^) which was predicted to prevent the formation of the first turn in the transition domain. In the second construct, we deleted the first four amino acids with the highest turn forming potential (SH^ΔWPGR^-SEAP^ΔSP^) of the predicted transition domain. In the third construct, we deleted the first four amino acids of the transition area and introduced additional substitutions in the remaining four amino acids in order to completely disrupt the transition area (SH^ΔWPGR/mut^-SEAP^ΔSP^) (for a schematic of all constructs, see Figure 2 B).

**Figure 5:**
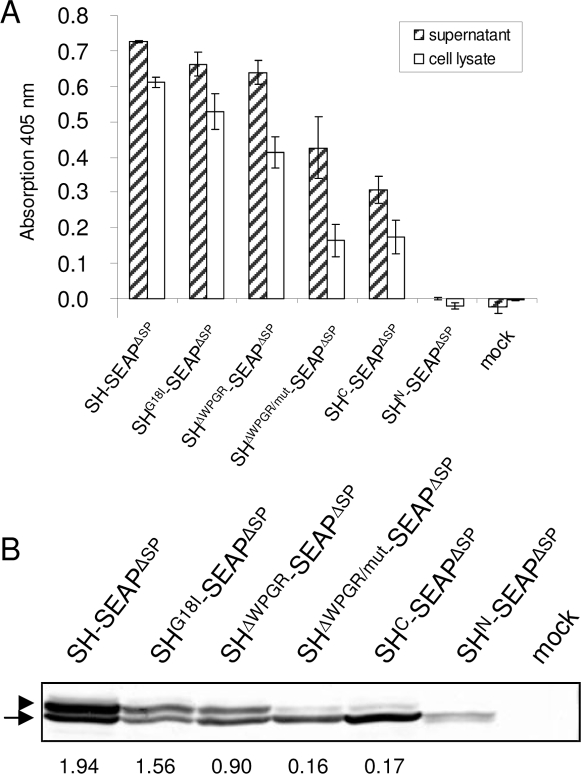
Mutation of the transition area impairs secretory activity of SEAP fusion proteins. (A) SEAP activity was measured in the supernatant (hatched bars) and whole cell lysate (white bars) of transfected HEK 293T cells after 5 min substrate incubation. Cells transfected with the empty vector were used as negative control (mock). Error bars show *s.e.m.* (*N* = 4). The data for cells with constructs SH^N^-SEAP^ΔSP^, SH^C^-SEAP^ΔSP^, SH-SEAP^ΔSP^ and mock are adopted from Figure 2A. (B) Western blots were prepared from whole cell lysates of transfected HEK 293T cells, and SEAP fusion proteins were detected with anti-myc antibody. The upper bands of the fusion proteins, except of that from SH^N^-SEAP^ΔSP^, represent the N-glycosylated and N-terminally processed protein population (▸), the lower band the non processed population (arrow). The values below the lanes show the density ratio of the upper band to the lower band for each fusion protein which decreases the more the transition area is impaired.

Each of these constructs was transfected into HEK 293T cells, and again SEAP activity was determined in the supernatants as well as in whole cell lysates. As shown in Figure 5A, SEAP activity decreases with increasing disruption of the transition area. SH^ΔWPGR/mut^-SEAP^ΔSP^ showed the lowest activity which is similar to the activity of SH^C^-SEAP^ΔSP^. This is consistent with the assumption that the transition area may be needed for the overall secretion activity of the shrew-1 signal sequence.

The dependency of secretion efficiency on the integrity of the transition area should be mirrored in the presence of N-glycosylated SEAP. This was tested by Western blotting (Figure 5B). With increasing impairment of the transition area the ratio of N-glycosylated (upper band, ▸) to non-glycosylated SEAP fusion protein (lower band, arrow) species decreased by one order of magnitude from 1.94 to 0.17 (Figure 5B). We conclude that protein export efficiency appears to be correlated with the existence and integrity of the transition area separating N- and C-domains of the shrew-1 signal peptide.

## Discussion

Here we report the first systematic approach for predicting structure and function of long signal peptides of single-pass integral membrane proteins. Sequence analysis tools suggest a general organization model for these sequences, which was validated in a proof-of-principle study using the type I membrane protein shrew-1. Most importantly, according to our NtraC model a structural feature of the transition area is a crucial determinant of long signal peptide modularization: A potentially turn- or loop-forming central element (transition area) acts as some kind of separation unit between two sequence domains with different targeting capacity. Results of cellular targeting studies highlight the functional importance of the transition area. A minimal interpretation is that it affects ER translocation of the reporter protein.

The N-domain (residues 1–19) was able to act as a mitochondrial targeting signal in our experiments. Similar observations have been made for other proteins containing consecutive “tandem” signals rather than “cryptic” signals as described by the NtraC model. The transmembrane glycoprotein nicastrin, which is an essential component of gamma-secretase [41], is such an example. Gamma-secretase was found to translocate into mitochondria in Alzheimer patients, potentially inducing apoptosis [42]. Transport into the organelle is mediated by a mitochondrial transit signal following the N-terminal cleavable signal peptide of nicastrin. Notably, in contrast to the shrew-1 example and the NtracC domain model, the sequential order of the targeting signals is inverted in nicastrin and other proteins containing such a “tandem” signal, *e.g.* microsomal CYP2E1 [43]. This demonstrates that the prediction and discovery of proteins with multiplex locations is important for an understanding of the regulation of cell process such as apoptosis.

Mitochondrial targeting of shrew-1 and other proteins containing NtraC-organized long signals may not occur constitutively but in a regulated manner or only under cellular stress, and our results indicate that the mitochondrial targeting signal (N-domain) and the ER targeting signal (C-domain) are not sequentially processed. The N-domain of shrew-1 harbors no ER translocation activity, but is able to mediate mitochondrial targeting. We wish to stress that this activity has been proven for the isolated N-domain in the context of the experimental setup used in the present study, and it needs further investigation to determine the conditions under which this activity is found in the context of the full-length signal peptide. Possibly this cryptic activity is revealed under certain physiological situations only.

As an extension to the already known tandem signals like in the nicastrin or CYP2E1 precursors [41], [43], our NtraC model provides a framework for cryptic signals. The domain model is of general relevance, as at least 62% of the known vertebrata proteins with a signal peptide exceeding 40 residues show an NtraC-organization. Although it remains unclear if and under which conditions or regulatory control mitochondrial targeting of these proteins occurs, we were able show that NtraC-organized signal peptides can exhibit additional functions besides ER targeting or protein export. Prediction of such important structural elements has now become feasible.

Due to its amphipathic nature, we further speculate that the N-domain might be involved in dimerization or stabilization of shrew-1 in the plasma membrane or interaction with other proteins [29], [32]. Positively charged arginine residues in the N-domain could help the signal peptide to adopt its native conformation in the plasma membrane. It would thereby follow the “positive inside rule” [44] and arrest the C-terminal part inside the membrane while being available for protein-protein interactions on the cytoplasmic side.

The C-domain is sufficient for protein export *via* the ER, but not as effective as the full-length signal peptide. Most strikingly, the transition area which was first predicted to only link the N- to C-domain, turned out to be essential for the full ER translocation activity of the C-domain. It is noteworthy that the transition area is the only part of the long signal peptides predicted to predominantly contain β-turns. Thus, turn formation seems to be not only a structural element separating the N- and C-domains, but a decisive feature of long signal peptides supporting the ER translocation activity of the C-domain. The NtraC model thereby explains earlier observations made for interleukin-15, which is subjected to different export rates depending on the length of its signal peptide [27].

Our model also provides a rational explanation for membrane targeting of bacterial autotransporters, which possess long signal peptides: These are in accordance with our NtraC model, where the C-domain alone is sufficient for transport to the inner membrane but for proper processing the complete signal peptide is required [45]. In the present study, we restricted our analysis to single-spanning integral membrane proteins with signals that have a similar organization as the long signal peptide of shrew-1. The role of the transition area besides making the N- and C-domain distinguishable is subject to further research.

## Materials and Methods

### Oligonucleotides used for cloning of SEAP fusion constructs

Constructs were generated by PCR (Suppl. Text S1).

### Cell lines, cell culture and transfection

HEK 293T (CRL-11268; ATCC, Manassas, USA) were cultured in Dulbecco's Modified Eagle Medium (DMEM; (Invitrogen GmbH, Karlsruhe, Germany) with 10% fetal calf serum (FCS; PAA LABORATORIES, Cölbe, Germany) and 1% penicillin/streptomycin (Invitrogen GmbH, Karlsruhe, Germany). 6×10^5^ cells were seeded per 12 cm^2^ of culture dish and transfected with 3 µg DNA 24 h later by using Magnet Assisted Transfection (MATra, IBA GmbH, Göttingen, Germany) according to the manufacturer's instructions.

### SEAP activity assays

SEAP activity assays were performed according to [39] using 10 µl of the supernatants or 6 µg of protein from cleared whole cell lysates.

### Immunoblotting and antibodies

After collection of supernatant for SEAP assays, cells were washed with PBS and lysed with 100 µl RIPA buffer (150 mM NaCl, 50 mM Tris-HCl, pH 7.5, 0.5% sodium deoxycholate, 1% Nonidet P-40, 0.1% SDS) containing proteinase inhibitor cocktail Complete (Roche Diagnostics GmbH, Mannheim, Germany) at 4°C for 30 min. Lysates were cleared by centrifugation in a microcentrifuge at 4°C for 5 min. Where indicated, cell lysates were treated with PNGase F which removes N-glycans according to the manufacturer's instructions (New England Biolabs, Frankfurt, Germany). For immunoblotting, 20 µg of protein from each cell lysate was separated in a 6% SDS PAA-gel. Protein blots were incubated with rabbit polyclonal anti-myc antibody (0.5 µg/ml; Sigma-Aldrich Chemie GmbH, München, Germany) diluted in TBST (10 mmol/L Tris-HCl, pH 7.4, 150 mmol/L NaCl; 0.05% Tween 20). Glyceraldehyde-3-phosphate dehydrogenase (GAPDH) was probed with mouse monoclonal anti-GAPDH antibody (1 µg/ml, Ambion/Applied Biosystems, Darmstadt, Germany), cytochrome c with mouse monoclonal anti-cytochrome c antibody (0.4 µg/ml; medac, Wedel, Germany) and Grp94 with rat monoclonal anti-Grp94 antibody (2 µg/ml; medac, Wedel, Germany). Secondary alkaline phosphatase-conjugated goat anti-rabbit antibody, horseradish peroxidase-conjugated goat anti-rabbit, horseradish peroxidase conjugated goat anti-mouse antibody and horseradish peroxidase conjugated goat anti-rat antibody (all Jackson ImmunoResearch, Dianova GmbH, Hamburg, Germany) were used for detection of first antibodies. Enzyme substrates were NBT/BCIP (Roche Diagnostics GmbH, Mannheim, Germany) for alkaline phosphatase or a solution of luminol (2.5 mM), p-coumaric acid (0.4 mM), Tris-HCl, pH 8.5 (100 mM) and 0.009% H_2_O_2_ for horseradish peroxidase.

### Densitometric analysis

The densitometric analysis of the Western blots was performed with Image J (Scion). The densities of the corresponding bands on the blot were measured and the ratio of the upper band to the lower band of each construct was calculated.

### Isolation of mitochondria

24 hours after transfection of HEK 293T cells mitochondria were isolated with the Qproteome Mitochondria Isolation Kit (Qiagen, Hilden, Germany) according to the manufacturer's instructions. Briefly, after removal of nuclei, cell debris, cytosolic and microsomal cell fractions, the mitochondria pellet was resuspended in 0.5 M sucrose buffer (1 mM EDTA, 0.1% BSA, 10 mM Tris-HCl, pH 7.5), layered on a 1–2 M sucrose gradient (1 mM EDTA, 0.1% BSA, 10 mM Tris-HCl, pH 7.5) and centrifuged for 2 h at 25,000 rpm. The mitochondrial band was collected, diluted with 2 volumes of 1 mM EDTA, 10 mM Tris-HCl, pH 7.4 buffer and pelleted by centrifugation at 20,000×g for 15 min. 20 µg of protein of each fraction was loaded on a 10% PAA-gel and separated by SDS-PAGE.

## Supporting Information

Table S1 Vertebrate signal peptides >40 amino acids, which are predicted to be NtraC organized but differ in their domain capacity from shrew-1. Underlined residues are predicted turns belonging to the T-domain
(0.17 MB DOC)

Figure S1 Multiple sequence alignment of the signal peptides of shrew-1 homologues
(0.37 MB DOC)

Text S1 Oligonucleotides used for cloning of SEAP fusion constructs.
(0.06 MB DOC)
