# Supplementary material for: Domain Organization of Long Signal Peptides of Single-Pass Integral Membrane Proteins Reveals Multiple Functional Capacity
Source: PLoS One. 2008 Jul 23;3(7):e2767. doi: 10.1371/journal.pone.0002767 (PMC2447879; doi:10.1371/journal.pone.0002767)
Supplement: Table S1 — Vertebrate signal peptides >40 amino acids, which are predicted to be NtraC organized but differ in their domain capacity from shrew-1. Underlined residues are predicted turns belonging to the T-domain (0.17 MB DOC) [file pone.0002767.s001.doc]

**Table S1**. Vertebrate signal peptides > 40 amino acids, which are predicted to be NtraC organized but differ in their domain capacity from shrew-1. Underlined residues are predicted turns belonging to the T-domain.

ID 33-45: signal peptides containing a predicted mTP coding N-Domain and a signal anchor encoding C-Domain

ID 46-174: signal peptides containing a signal peptide encoding C-Domain

ID 175-185: signal peptides containing a signal anchor encoding C-Domain

| ID | NCBI Accession Number | Signal peptide sequence |
| --- | --- | --- |
| 33 | Q60813 | MSVAAAGRGFASSLSSPQIRRIALKEAKLTPHIWAALHWNLGLRLVPSVRVGILVLLIFLPSTFC |
| 34 | P08607 | MCAKQQQTLLPTRAAHGRLHRNRDVVAWPFSTLCRVSGPTLFQMTFTAALWVAVFG |
| 35 | P61569 | MNPSEMQRKAPPRRRRHRNRAPSSHKMNKMMMSEEQMKLPSTNKAEPLTWAQLNKLTQLATKCLENTKMTQTPESMLLAALMIVSTVVS |
| 36 | P61570 | MNPSEMQRKAPPRRRRHRNRAPLTHKMNKMVTSEEQMKLPSTKKAEPPTWAQLKKLTQLATKYLENTKVTQTPESMLLAALMIVSMVVS |
| 37 | Q6ZVY0 | MNPSEMQGKAPPQRQRTRNRTSLTRRVNKMVISEEQMKLPSTKKAGPPTWAQLKKLTQLAEKSLENTRVTQTPENKLLAALMIVSTVVS |
| 38 | P61565 | MHPSEMQRKAPPRRRRHRNRAPLTHKMNKMVTSEQMKLPSTKKAEPPTWAQLKKLTQLATKYLENTKVTQTPESMLLAALMIVSMVVS |
| 39 | Q69384 | MNPSEMQRKAPPRRRRHRNRAPLTHKMNKMVTSEEQMKLPSTKKAEPPTWAQLKKLTQLATKYLENTKVTQTPESMLLAALMIVSMVVS |
| 40 | O71037 | MNPSEMQRKAPPRRRRHRNRAPLTHKMNKMVTSEEQMKLPSTKKAEPPTWAQLKKLTQLATKYLENTKVTQTPESMLLAALMIVSMVVS |
| 41 | Q9UKH3 | MNPSEMQRKAPPRRRRHRNRAPLTHKMNKMVTSEEQMKLPSTKKAEPPTWAQLKKLTQLATKYLENTKVTQTPESMLLAALMIVSMVVS |
| 42 | Q902F9 | MNPSEMQRKAPPRRRRHRNRAPLTHKMNKMVTSEEQMKLPSTKKAEPPTWAQLKKLTQLATKYLENTKVTQTPESMLLAALMIVSMVVS |
| 43 | Q902F8 | MNPSEMQRKAPPRRRRHRNRAPLTHKMNKMVTSEEQMKLPSTKKAEPPTWAQLKKLTQLATKYLENTKVTQTPESMLLAALMIVSMVVS |
| 44 | Q9UKH7 | MNPSEMQRKAPPRRRRHCNRAPLTHKMNKMVTSEEEMKLPSTKKAEPLTWAQLKKLTQLATKYLENTKVTQTPESMLLAALMIVSMVVS |
| 45 | Q9UBN6 | MGLWGQSVPTASSARAGRYPGARTASGTRPWLLDPKILKFVVFIVAVLLPVRVDS |
| 46 | O75077 | MKPPGSSSRQPPLAGCSLAGASCGPGRGPAGSVPASAPARTPPCRLLLVLLLLPPLAAS |
| 47 | Q9R1V7 | MKPPGSISRRPTLTGCSLPGASCGPGRCPAGPVPARAPPCRLLLVLLLLPALATS |
| 48 | P33727 | MGRRGAASLPRPGSPRRPLLPGVLPLLLRLLLLPSRPGAGA |
| 49 | P50429 | MGKLSPCTGRSRPGGPGPQLPLLLLLLQLLLLLLSPARASG |
| 50 | Q5FYB0 | MAPRGCAGHPPPPSPQACVCPGKMLAMGALAGFWILCLLTYGYLSWGQA |
| 51 | Q8BM89 | MAPRDSAEPLPPLSPQAWAWSGKFLAMGALAGFSVLSLLTYGYLCWG |
| 52 | Q8TE60 | MECALLLACAFPAAGSGPPRGLAGLGRVAKALQLCCLCCASVAAALA |
| 53 | Q4VC17 | MECALLCLCALRAAGPGPPWGPAGLGRLAKALQLCCFCCASVAVALA |
| 54 | Q9UHI8 | MQRAVPEGFGRRKLGSDMGNAERAPGSRSFGPVPTLLLLAAALLAVSDA |
| 55 | P97857 | MQPKVPLGSRKQKPCSDMGDVQRAARSRGSLSAHMLLLLLASITMLLC |
| 56 | Q9WUQ1 | MQPEVPLGSGKLKPCSDMGDIQRAAKFRSSQSAHMLLLLLASITMLLCVRGAHG |
| 57 | Q9TT93 | MSHMDSHPGRGLADGWLWGIQPRLLLPTVPVSGSRLVWLLLLASLLPSAWP |
| 58 | O75173 | MSQTGSHPGRGLAGRWLWGAQPCLLLPIVPLSWLVWLLLLLLASLLPSARL |
| 59 | Q8BNJ2 | MSQMGLHPRRGLTGHWLQRFQPCLPLHTVQWRRLLLLAFLLSLAWPASP |
| 60 | Q5RFQ8 | MSQTGSHPGRGLAGRWLWGAQPCLLLPIVPLSWLVWLLLLLLASLLPSARL |
| 61 | Q2VLG4 | MSKLRMVLLEDSGSADVRRHFVNLSPFTIAVVLLLRACFVTSSLG |
| 62 | Q2VL90 | MDKLRMVLHENSGSADFRRCSAHLSSFTFAVVAVLSACLVTSSLGG |
| 63 | Q9NR16 | MMLPQNSWHIDFGRCCCHQNLFSAVVTCILLLNSCFLISS |
| 64 | Q9BY67 | MASVVLPSGSQCAAAAAAAAPPGLRLRLLLLLFSAAALIPTGDG |
| 65 | Q8R5M8 | MASAVLPSGSQCAAAAAVAAAAAPPGLRLRLLLLLLSAAALIPTGDG |
| 66 | Q5RBP6 | MGVKAAQTGIWASQQQSIRVVGFQAQTAHRAICLLGFVVLVLLQCCSA |
| 67 | P17927 | MGASSPRSPEPVGPPAPGLPFCCGGSLLAVVVLLALPVAWG |
| 68 | Q8AWW5 | MYLAAVSAGRRRPGGDGGGGGGGWHLAAAGWLLLLALLLGQPGTRA |
| 69 | Q9Y426 | MAMARLGSWLGEAQWLALVSLFVAALATVGLYLAQWALARA |
| 70 | Q05754 | MQPHLSHQPCWSLPSPSVREAASMYGTAVAIFLVILVAALQG |
| 71 | Q9H6D8 | MPSGCHSSPPSGLRGDMASLVPLSPYLSPTVLLLVSCDLGFVRA |
| 72 | Q5SZK8 | MHSAGTPGLSSRRTGNSTSFQPGPPPPPRLLLLLLLLLSLVSRVPA |
| 73 | O57328 | MAERRGPAGGGSGEVGGRRAGGDRCPRRPPALPLLLLLWAAALPAGG |
| 74 | Q9UP38 | MAEEEAPKKSRAAGGGASWELCAGALSARLAEEGSGDAGGRRRPPVDPRRLARQLLLLLWLLEAPLLLG |
| 75 | O70421 | MAEEAAPSESRAAGRLSLELCAEALPGRREEVGHEDTASHRRPRADPRRWASGLLLLLWLLEAPLLLG |
| 76 | Q08463 | MAEEAVPSESRaagrPSLELCAVAlPGRREEVGHQDTAGHRRPRAHSRCWARGLLLLLWLLEAPLLLG |
| 77 | Q5ZLR1 | MAGAIIENMSTRKLCIVGGILLVFQVIAFLVGGLIAPSPTTA |
| 78 | Q5T9L3 | MAGAIIENMSTKKLCIVGGILLVFQIIAFLVGGLIAPGPTTA |
| 79 | Q6DID7 | MAGAIIENMSTKKLCIVGGILLVFQIVAFLVGGLIAPAPTTA |
| 80 | Q5R9R3 | MAGAIIENMGTKKLCIVGGILLVFQIIAFLVGGLIAPGPTTA |
| 81 | Q6P689 | MAGAIIENMSTKKLCIVGGILLVFQIVAFLVGGLIAPAPTTA |
| 82 | P55203 | MTACTFLAGGLRDPGLCGPTRWAPSPPGLPPIPPRPRLRLRPPLLLLLLLPRSVLS |
| 83 | O19179 | MSACALLAGGLPDPRLCAPARWARSPPGVPGAPPWPQPRLRLLLLLLLLPPSALS |
| 84 | Q02846 | MTACARRAGGLPDPGLCGPAWWAPSLPRLPRALPRLPLLLLLLLLQPPALS |
| 85 | P51839 | MAGLQQGCHPEGQDWTAPHWKTCRALPGPRGLTVRHLRTVSSISVFSVVFWGVLLWADSLSLPAWA |
| 86 | O02740 | MFLAPWPFSHLMLWFVTLGRQRGQHGLASFKLLWCLWLLVLMSLPL |
| 87 | Q5SDA5 | MFLGPWPFSRLLSWFAISSRLSGQHGLPSSKFLRCLCLLALLPLLRWGQA |
| 88 | P51842 | MFLGPWPFSRLLSWFAISSRLSGQHGLTSSKFLRYLCLLALLPLIWWGQA |
| 89 | Q6TL19 | MASRTRSESPLEPRLYAGAGSRADHPSLVLMLSVVMLVTCLEA |
| 90 | P55205 | MASRARSEPPLEHRFYGGAESHAGHSSLVLTLFVVMLMTCLEA |
| 91 | Q91ZJ9 | MLGLTQHAQKVWRMKPFSPEVSPGSSPATAGHLLRISTLFLTLLELAQVCRG |
| 92 | Q9JHJ8 | MQLKCPCFVSLGTRQPVWKKLHVSSGFFSGLGLFLLLLSSLCAASA |
| 93 | Q01113 | MGLGRCIWEGWTLESEALRRDMGTWLLACICICTCVCLGV |
| 94 | Q00651 | MFSTKSAWLRNGGADQGPRGIALREAVMLLLYFGVPTGPS |
| 95 | Q6UXG2 | MAEPGHSHHLSARVRGRTERRIPRLWRLLLWAGTAFQVTQG |
| 96 | Q8NC54 | MAAAVPKRMRGPAQAKLLPGSAIQALVGLARPLVLALLLVSAALSSVVS |
| 97 | Q9QYN3 | MRRLKSDWKLSTETREPGARPALLQARMILRLIALALVTGHVGG |
| 98 | Q61001 | MAKRGGQLCAGSAPGALGPRSPAPRPLLLLLAGLALVGEA |
| 99 | Q2KIY5 | MVAPMYGSPGGRLARAVTRALALALVLALLVGLFLSGLTGA |
| 100 | P42703 | MAAYSWWRQPSWMVDNKRSRMTPNLPWLLSALTLLHLTMHANG |
| 101 | O70535 | MGAFSWWRQPSWMADNKRGRMTPSLPWLLSALTLLHLMMHVNG |
| 102 | P49256 | MAAEGWIWRWGWGRRCLGRPGLPGPGPGPATPLFLLLLLGPVVA |
| 103 | Q12907 | MAAEGWIWRWGWGRRCLQRPGLLGPGPGPTTPLFLLLLLGSVTA |
| 104 | Q9DBH5 | MAAEAWLWRWGWGWGQRCPGRPGLPGPGPSPTTFLHLLLLLGPVAA |
| 105 | Q6ZSA7 | MGSLQHCCCLLPKMGDTWAQLPWPGPPHPAMLLISLLLAAGLMHSDA |
| 106 | Q3UY51 | MGSLQHCCCQLPKMGDTWAQLPWPGPPHSALLLVFFLLAAGVMHSDA |
| 107 | Q4KLL3 | MGSLQYCCCQLPKMGDTWAQLPWPGPPHSALLLVFFLLAAGVMHSDA |
| 108 | Q9NS15 | MPGPRGAAGGLAPEMRGAGAAGLLALLLLLLLLLLGLGGRVEG |
| 109 | Q29451 | MVGDARPSGVRAGGCRGAVGSRTSSRALRPPLPPLSSLFVLFLAAPCAWA |
| 110 | Q8VHC8 | MGASVLPLGLGAGDCQSSSGRRMSACLPRTALSFLLSLLLATPGARA |
| 111 | O46432 | MGADARPLGVRAGGGGRGAARPGTSSRALPPPLPPLSFLLLLLAAPGARA |
| 112 | O00754 | MGAYARASGVCARGCLDSAGPWTMSRALRPPLPPLCFFLLLLAAAGARA |
| 113 | O09159 | MGTGPLTSGVRAGGGNTGWLWMSSCNLGSPVLPISFLFWLLLAAPGARA |
| 114 | Q641Q3 | MRGAARAAWGRAGQPWPRPPAPGPPPPPLPLLLLLLAGLLGGAGA |
| 115 | Q9Y5R2 | MPRSRGGRAAPGPPPPPPPPGQAPRWSRWRVPGRLLLLLLPALCCLPGAARA |
| 116 | P08169 | MEAAAGRSSHLGPAPAGRPPRCPLLLQLQLLLLLLLLPPGWVPG |
| 117 | Q9IAL7 | MALCKKTVGSVLEEWCLNEPLFGCKRHQNVRKKLRLIRIIGLLVSVVAISTFSLSISA |
| 118 | Q9UI40 | MDLQQSTTITSLEKWCLDESLSGCRRHYSVKKKLKLIRVLGLFMGLVAISTVSFSISA |
| 119 | O54701 | MDLHQSATVRLLQEWCSHESPSGCRRHYNTRKKLKLIRVIGLVMGLVAVSTVPFSISA |
| 120 | Q969V3 | MLEEAGEVLENMLKASCLPLGFIVFLPAVLLLVAPPLPAADA |
| 121 | Q8VCM8 | MLEEAGEVLENVLKASCLPLGFIVFLPAVLLLVAPPLPAADA |
| 122 | Q5XIA1 | MLEEAGEVLENVLKASCLPLGFIVFLPAVLLLVAPPLPAADA |
| 123 | Q99519 | MTGERPSTALPDRRWGPRILGFWGGCRVWVFAAIFLLLSLAASWSKA |
| 124 | O35657 | MVGADPTRPRGPLSYWAGRRGQGLAAIFLLLVSAAESEARA |
| 125 | Q8NET5 | MENQPVRWRALPGLPRPPGLPAAPWLLLGVLLLPGTLRLAGG |
| 126 | Q9HCJ2 | MLNKMTLHPQQIMIGPRFNRALFDPLLVVLLALQLLVVAGLVRA |
| 127 | Q8C031 | MLNKMTLHPQQIMIGPRFNRALFDPLLVVLLALQLLVVAGLVRA |
| 128 | Q8N2Q7 | MALPRCTWPNYVWRAVMACLVHRGLGAPLTLCMLGCLLQAGHVLS |
| 129 | Q99K10 | MALPRCMWPNYVWRAMMACVVHRGSGAPLTLCLLGCLLQTFHVLS |
| 130 | Q62765 | MALPRCMWPNYVWRAMMACVVHRGSGAPLTLCLLGCLLQTFHVLS |
| 131 | Q8K1M5 | MDLSALASSREVRGPGPGAPVNRPLLPLLLLLLLLPLPASA |
| 132 | Q28142 | MYQRMLRCGAELGSPGGGGGGGGRLALLWIVPLTLSGLLGVAWG |
| 133 | P58400 | MYQRMLRCGAELGSPGGGGGGGGGGGAGGRLALLWIVPLTLSGLLGVAWG |
| 134 | Q63373 | MYQRMLRCGAELGSPGGGSSGGAGGRLALLWIVPLTLSGLLGVAWG |
| 135 | Q96JQ0 | MQKELGIVPSCPGMKSPRPHLLLPLLLLLLLLLGAGVPGAWG |
| 136 | Q63415 | MPPRAPPAPGPRPPPRAAGRHGLSPLAPRPWRWLLLLALPAVCSA |
| 137 | P05164 | MGVPFFSSLRCMVDLGPCWAGGLTAEMKLLLALAGLLAILATPQPSEG |
| 138 | Q63259 | MRRPRRPGGPAGCGGSEGSGGLRLLVCLLLLSGRPGGCSA |
| 139 | Q8BQC3 | MAEPRTASPRRLPALRRPGFLPPLLPPPPPPLLLLLLLLPLPAPSLG |
| 140 | Q9JLB9 | MARTPGPAPLCPGGGKAQLSSAFPPAAGLLLPAPTPPPLLLLLIPLLLFSRLCGALA |
| 141 | Q8JGM4 | MWRRRARSGGGGGGGGGGAAPRCRWWPAVLALLAAALPAARS |
| 142 | O60895 | MASLRVERAGGPRLPRTRVGRPAALRLLLLLGAVLNPHEALA |
| 143 | Q9WUP0 | MAPLRVERAPGGSRLGVTRAQRPTALCLPPLLLLLLLLLGAVSA |
| 144 | Q9JHJ1 | MAPLRVERAPGGSQLAVTSAQRPAALRLPPLLLLLLLLLLGAVST |
| 145 | Q6NW40 | MGLRAAPSSAAAAAAEVEQRRRPGLCPPPLELLLLLLFSLGLLHA |
| 146 | Q7TQ33 | MGVRAAPSCAAAPAAAGAEQSRRPGLWPPSPPPPLLLLLLLSLGLLHA |
| 147 | P50228 | MSLQLRSSAHIPSGSSSPFMRMAPLAFLLLFTLPQHLAEA |
| 148 | Q64519 | MKPGPPRRGTAQGQRVDTATHAPGARGLLLPPLLLLLLAGRAAG |
| 149 | P33671 | MKPGPPRRGTAQGQRVDTATHGPGARGLLLPPLLLLLLAGRAAG |
| 150 | Q8AV58 | MVGRKVDREIIARRNSRRDGMMMKLNFCFFFCRRWWAFLLLQLHMLQALA |
| 151 | Q9W6G6 | MKTAGEPDRRRQRRQVRTGRFSCAWWSTSVMLFFSLPEGNC |
| 152 | Q9Z123 | MLARAERPRPGPRPPPVSLFPPPSSLLLLLLAMLSAPVCG |
| 153 | Q96PQ0 | MAHRGPSRASKGPGPTARAPSPGAPPPPRSPRSRPLLLLLLLLGACGAAG |
| 154 | Q9EPR5 | MAHRGPPSAPKRPGPTAPDRSFQALLPPCWPRSWPLLLLLLVLVAACGA |
| 155 | Q96GP6 | MEGAGPRGAGPARRRGAGGPPSPLLPSLLLLLLLWMLPDTVAP |
| 156 | Q9UIK5 | MVLWESPRQCSSWTLCEGFCWLLLLPVMLLIVARPVKLAA |
| 157 | Q9QYM9 | MVLWESPRQCSSWTLCEGFCWLLLLPVTLLIIARPVKLAA |
| 158 | Q6R5N8 | MSGLYRILVQLEQSPYVKTVPLNMRRDFFFLVVTWMPKTVKMNGSSFVPSLQLLLMLVGFSLPPVAET |
| 159 | Q6ZP80 | MRLNIAIFFGALFGALGVLLFLVAFGSDYWLLATEVGRCSG |
| 160 | O14763 | MEQRGQNAPAASGARKRHGPGPREARGARPGPRVPKTLVLVVAAVLLLVSAESAL |
| 161 | Q9QZM4 | MEPPGPSTPTASAAARADHYTPGLRPLPKRRLLYSFALLLAMLQAVFVPVTA |
| 162 | P49744 | MTMITPSSKLTLTKGNKSWSSTRCGAFLLLHLVLQPWQRAGA |
| 163 | O95185 | MRKGLRATAARCGLGLGYLLQMLVLPALALLSASGTGSAA |
| 164 | O08747 | MRKGLRATAARCGLGLGYLLQMLVLPALALLSASGTGSAA |
| 165 | Q761X5 | MRKGLRATAARCGLGLGYLLQMLVLPALALLSASGTGSAA |
| 166 | Q15904 | MMAAMATARVRMGPRCAQALWRMPWLPVFLSLAAAAAAAAA |
| 167 | P98165 | MRSSRQRGDRSAATGGGCGARRWALPRCGALCLLLALGCLRTA |
| 168 | P31286 | MRKNLWTFQFGGSGLVGSAMVSQHFVVLLMSLYCLTQS |
| 169 | Q9ULT6 | MRPRSGGRPGATGRRRRRLRRRPRGLRCSRLPPPPPLPLLLGLLLAAAGPGAARA |
| 170 | Q5SSZ7 | MRPRSGGRPGAPGRRRRRLRRGPRGRRLPPPPPLPLLLGLLLAAAGPGAARA |
| 171 | Q9BS86 | MEAFALGPARRGRRRTRAAGSLLSRAAILLFISAFLVRVPSSVG |
| 172 | Q62522 | MEALAPGRAPRGRRRAGASGSVLSPLSLAAVLLCALLRAPPAVG |
| 173 | Q2YHT5 | MEISQQAGWCKKPASPMNTRAALEAVRNTAWTIVLLTSAAVMGAS |
| 174 | Q90YI0 | MNFTEGCEATGRRPGSAGSRRRRAPRPGPVALLPLLLPLLLPPAAAV |
| 175 | Q0VD19 | MPRHGVSPGQGLPRSGREQASDRSLGAPCLRLLWLGLALA |
| 176 | Q04519 | MPHHRASSGQDHLRAGWEQRLERSLPAPRVGLLWMGLGLALVLA |
| 177 | Q86VB7 | MSKLRMVLLEDSGSADFRRHFVNLSPFTITVVLLLSACFVT |
| 178 | Q28065 | MKHQRVPVMILHSKGTMASWPFSRLWSISDPILFQVTLVATLLATVLG |
| 179 | P60509 | MDPLHTIEKVPARRNIHDRGHQGHRMGDGTPGRPKISVQQMTRFSLIIFFLSAPFVVNA |
| 180 | P42702 | MMDIYVCLKRPSWMVDNKRMRTASNFQWLLSTFILLYLMNQVNS |
| 181 | P51511 | MGSDPSAPGRPGWTGSLLGDREEAARPRLLPLLLVLLGCLG |
| 182 | Q5VX71 | MYHGMNPSNGDGFLEQQQQQQQPQSPQRLLAVILWFQLALC |
| 183 | Q8BH32 | MYHGMNPSNGDGFLEQQLQQQQPQSPQRLLAVILWFQLALC |
| 184 | Q5R8M2 | MYHGMNPSNGDGFLEQQQQQQPQSPQRLLAVILWFQLALC |
| 185 | Q8WTU2 | MHKEAEMLIGPQLDEKRWGWRLGGDSAAPPFLPQALSFLLLLPL |
