# Supplementary material for: Domain Organization of Long Signal Peptides of Single-Pass Integral Membrane Proteins Reveals Multiple Functional Capacity
Source: PLoS One. 2008 Jul 23;3(7):e2767. doi: 10.1371/journal.pone.0002767 (PMC2447879; doi:10.1371/journal.pone.0002767)
Supplement: Figure S1 — Multiple sequence alignment of the signal peptides of shrew-1 homologues (0.37 MB DOC) [file pone.0002767.s002.doc]

**Figure S1.** Multiple sequence alignment of the signal peptides of shrew-1 from *Homo sapiens, Macaca mulatta, Canis familaris, Bos taurus, Mus musculus, Rattus norvegicus*, and *Danio rerio* using ClustalW (version 1.83)[[1]](#footnote-2). Shaded in light gray: N-domain, shaded in dark gray: C-domain. Underlined areas are predicted turn areas. The black triangle marks the signal peptidase cleavage side predicted by SignalP for *H. sapiens* shrew-1 (cleavage site probability: 0.88). The black frame marks the predicted transition areas.

Identity (in %) between the human transition area (19 residues: WPGRPLGSH) and the vertebrate homologues. The observed number of substitutions is given in brackets: *M. mulatta* 100% (0), *C. familaris* 89% (2), *B. taurus* 89% (2), *M. musculus* 95% (1), *R. norvegicus* 95% (1), *D. rerio* 74% (5). The two glycine residues at positions 18 and 22, and leucine at position 21 are conserved over all species, pointing to the importance of this position.

1. a) Chenna, R. *et al.*, Multiple sequence alignment with the Clustal series of programs. *Nucl. Acids Res.* **31**, 3497-3500 (2003); b) Labarga, A., Valentin, F., Andersson, M., Lopez R. Web services at the European Bioinformatics Institute. *Nucl. Acids Res.* Web Services Issue (2007). [↑](#footnote-ref-2)
