# Supplementary material for: Domain Organization of Long Signal Peptides of Single-Pass Integral Membrane Proteins Reveals Multiple Functional Capacity
Source: PLoS One. 2008 Jul 23;3(7):e2767. doi: 10.1371/journal.pone.0002767 (PMC2447879; doi:10.1371/journal.pone.0002767)
Supplement: Text S1 — Oligonucleotides used for cloning of SEAP fusion constructs. (0.06 MB DOC) [file pone.0002767.s003.doc]

**Oligonucleotides used for cloning of SEAP fusion constructs.**

Restriction sites used for cloning into pcDNA3.1(-) (Invitrogen, Karlsruhe, Germany) are underlined.

NotI SH^C^-SEAP^ΔSP^:

5’-TTGCGGCCGCATGCCCCTCGGAAGCCATGCCTGG-3’.

NotI SH^N^-SEAP^ΔSP^:

5’-TGCGGCCGCATGTGGATTCAACAGCTTTTAGGACTCAGCTCCATGT

CCATCCGCTGGCCGGGCCGCGAATTCATCATCCCAGTTGAG-3’.

NotI SH^G18I^-SEAP^ΔSP^:

5’- GCGGCCGCATGTGGATTCAACAGCTTTTAGGACTCAGCTCCATGTC

CATCCGCTGGCCGATCCGCCCCCTCGGAAGCC-3’.

NotI SH^ΔWPGR^-SEAP^ΔSP^:

5’-TTGCGGCCGCATGTGGATTCAACAGCTTTTAGGACTCAGCTCCATGT

CCATCCGCCCCCTCGGAAGCCATGCCTGG-3’.

NotI SH^ΔWPGR/mut^-SEAP^ΔSP^:

5’-TTGCGGCCGCATGTGGATTCAACAGCTTTTAGGACTCAGCTCCATGT

CCATCGCCCCCTCGGACCATGCCTGG-3’.

Acc65I SEAP Myc-tagging:

5’-TTGGTACCTTACAGATCCTCTTCTGAGATGAGTTTTTGTTCACCCGG

GTGCGCGGCGTCG-3’.

SH-SEAP^ΔSP^ served as a template. It was generated by fusion of the shrew-1 signal peptide amplified by PCR with the primers

NotI shrew-1 SP 5’-TTTGCGGCCGCATGTGGATTCAACAGCTT-3’,

and EcoRI shrew-1 SP 5’-TTGAATTCGCCCAGGGCCTCGCAGGC-3’ and SEAP, lacking the endogenous signal peptide, amplified by PCR with the following primers:

EcoRI 5’-TTGAATTCATCATCCCAGTTGAGGAG-3’, and HindIII 5’-TTTTAAGCTTTTAACCCGGGTGCGCGGC-3’ at the EcoRI restriction site.

Myc-tagging of the construct was done with the primer Acc65I SEAP Myc-tagging

5’-TTGGTACCTTACAGATCCTCTTCTGAGATGAGTTTTTGTTCACCCGGG

TGCGCGGCGTCG-3’.

SEAP^ΔSP^ was generated with the primers XhoI start codon SEAP^ΔSP^

5’-TTTCTCGAGATGATCATCCCAGTTGAGGAG-3’ and

HindIII 5’-TTTTAAGCTTTTAACCCGGGTGCGCGGC-3’.

Myc-tagging of the construct was done with the primer Acc65I SEAP Myc-tagging

5’-TTGGTACCTTACAGATCCTCTTCTGAGATGAGTTTTTGTTCACCCGGGT

GCGCGGCGTCG-3
